# Case Report: Identification of a Novel Pathogenic Germline *TP53* Variant in a Family With Li–Fraumeni Syndrome

**DOI:** 10.3389/fgene.2021.734809

**Published:** 2021-09-01

**Authors:** Francesco Paduano, Fernanda Fabiani, Emma Colao, Francesco Trapasso, Nicola Perrotti, Vito Barbieri, Francesco Baudi, Rodolfo Iuliano

**Affiliations:** ^1^Medical Genetics Unit, University “Magna Graecia”, Catanzaro, Italy; ^2^Department of Health Sciences, University “Magna Graecia”, Catanzaro, Italy; ^3^Tecnologica Research Institute and Marrelli Health, Biomedical Section, Stem Cells and Medical Genetics Units, Crotone, Italy; ^4^Department of Experimental and Clinical Medicine, University Magna Graecia of Catanzaro, Catanzaro, Italy; ^5^Medical Oncology Unit, Mater Domini Hospital, Catanzaro, Italy

**Keywords:** Li-Fraumeni, TP53, targeted sequencing, LFS cancers, germline TP53 variant

## Abstract

Li–Fraumeni syndrome (LFS) is an inherited autosomal dominant disease characterized by a predisposition to many cancers. Germline pathogenic variants in *TP53* are primarily responsible for LFS. By performing a targeted sequencing panel in a proband with liver carcinoma having a deceased son affected by osteosarcoma, we found the novel heterozygous frameshift variant c.645del (p.Ser215Argfs^*^32) in the *TP53* gene. This variant co-segregated with typical LFS cancers in the family pedigree, consistent with the pathogenicity of this novel and previously undescribed *TP53* variant.

## Introduction

Li–Fraumeni syndrome (LFS) is a rare autosomal dominant disorder in which patients have an increased susceptibility to developing several childhood- and adult-onset tumors compared with other cancer syndromes (McBride et al., 2014; Schneider et al., 2019). LFS is usually associated with a family history of multiple malignancies, mainly the so-called core LFS cancers, including osteosarcomas, soft-tissue sarcomas, brain tumors, adrenocortical carcinomas, and early-onset female breast cancers (Egan et al., 2012; Zhou et al., 2017). Recent studies show that LFS is also associated with an increased risk of several additional cancers, including liver, lung, prostate, ovarian, and pancreatic gastrointestinal cancers; leukemia; and lymphoma as well as cancers of the kidney, larynx, lung, skin, testis, and thyroid (McBride et al., 2014; Schneider et al., 2019). Therefore, LFS is associated with the development of tumors at various sites compared with other hereditary syndromes that predispose subjects to cancer, commonly limited to specific tumor sites (McBride et al., 2014; Young et al., 2019).

LFS is mainly associated with germline variants in the *TP53* gene, a tumor-suppressor gene whose protein product is a transcription factor involved in several cell functions such as DNA repair, cell cycle arrest, and senescence as well as autophagy, apoptosis, and necrosis (Bougeard et al., 2015; Miller et al., 2016).

Importantly, p53 protein works not only as a transcriptional regulator, but can also act in the cytosol and mitochondria to promote apoptosis through transcription-independent mechanisms (Barnoud et al., 2021).

Because wild-type p53 protein works as a tumor suppressor, variants in the tumor-suppressor gene *TP53* that disrupt protein function or stability could be responsible for the accumulation of genomic alterations, culminating in the development of tumors, especially LFS-related cancers (Zhou et al., 2017; Di Agostino et al., 2019).

In addition to the loss of function that a mutation in *TP53* may cause, certain mutant forms of p53 can exert dominant-negative effects over the coexpressed WT p53 allele, whereas others can exert additional oncogenic activity by a gain-of-function mechanism (Barnoud et al., 2021).

Heterozygous *TP53* germline variants are reported in several families affected by LFS (Egan et al., 2012; Valdez et al., 2017; Kharaziha et al., 2019). Subjects who are carriers of a pathogenic *TP53* variant possess a variable lifetime risk of developing cancer, and the phenotype can differ from cancer-free over a lifetime to fully penetrant, depending on the functional and structural effects of the causative *TP53* variant (Olivier et al., 2010; de Andrade et al., 2017; Bittar et al., 2019). Here, we report a novel and rare heterozygous *TP53* germline pathogenic variant (c.645del) found in the proband, his son, and three relatives from one non-consanguineous south Italian family having an aggregated history of typical LFS cancers.

## Results

### Case Report and Genetic Analysis

**Figure 1:**
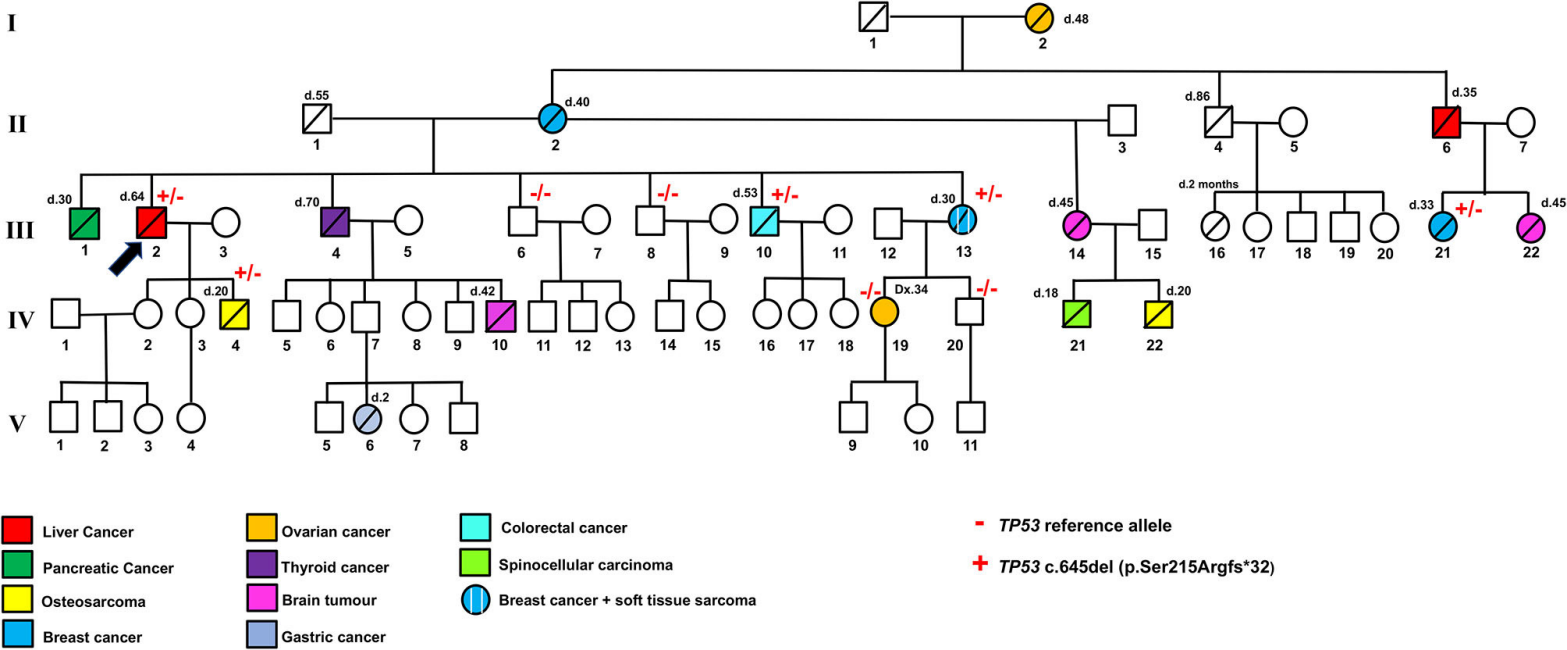
Family pedigree indicating the genotype and phenotype of each individual. + and – indicate the *TP53* pathogenic variant c.645del (p.Ser215Argfs*32) and *TP53* reference allele, respectively. Different colors indicate liver cancers, pancreatic cancer, osteosarcoma, breast cancer, ovarian cancer, thyroid cancer, brain tumor, gastric cancer, colorectal cancer, spinocellular carcinoma, and soft tissue sarcoma. d, age of death; Dx, age of diagnosis.

**Figure 2:**
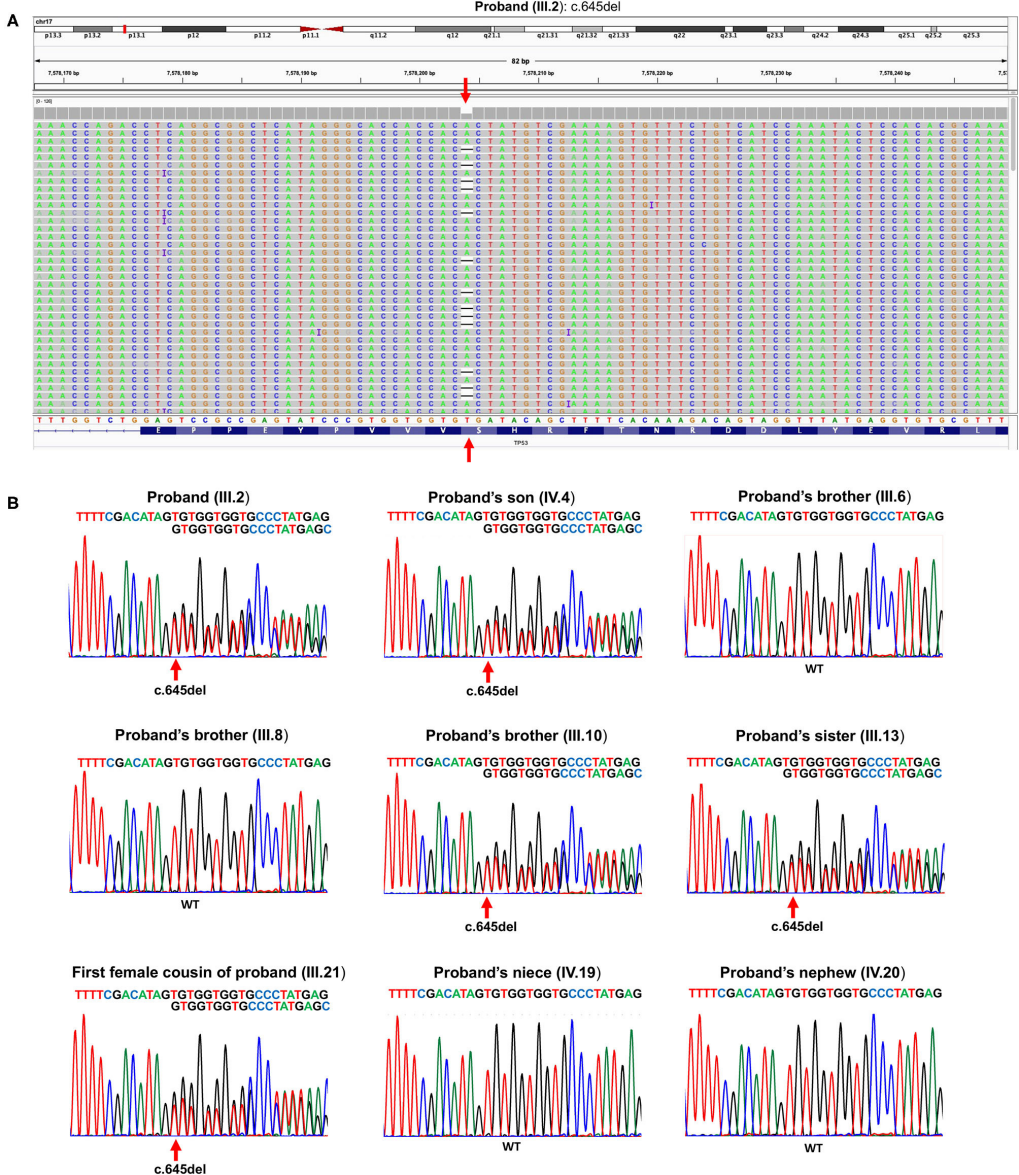
**(A)** Snapshot of Integrative Genomics Viewer, showing the *TP53* germline variant c.645del (p.Ser215Argfs*32) in the proband (III.2). **(B)** Sanger sequencing showing the c.645del pathogenic heterozygous *TP53* variant in the proband (III.2), his son (IV.4), his brother (III.10), his sister (III.13), and his first female cousin (III.21). WT *TP53* allele is shown in proband's brother (III.6), proband's brother (III.8), proband's niece (IV.19), and proband's nephew (IV.20). The red arrow indicates the heterozygous deletion.

The proband (III.2) is a 64-year-old deceased male affected by liver cancer, having two healthy daughters and one 20-year-old deceased son affected by osteosarcoma (Figure 1). Because of this latter finding as well as an aggregated family cancer history suggestive of p53 dysfunction, we analyzed the proband with a next-generation sequencing (NGS) tumor panel containing the *TP53, BRCA2, BRCA1, ATM, CHEK2*, and *PALB2* genes (Supplementary Methods). NGS analysis showed a heterozygous frameshift *TP53* variant ([GRCh37/hg19] chr17: g.7578204delA; NM_000546.6: c.645del (p.Ser215Argfs^*^32) in the exon 6 of the *TP53* (III.2, Figure 2A). Sanger sequencing confirmed the presence in the proband of the heterozygous frameshift c.645del variant in the exon 6 of the *TP53* gene (Figure 2B). No genetic alterations were detected within the other genes included in the panel.

Following this *TP53* mutation assessment, genetic counseling was also extended to proband family members, and several first- and second-degree relatives affected by tumors specifically associated with LFS were reported. Importantly, the proband's son (IV.4, Figure 2B), deceased at the age of 20 due to osteosarcoma, was a heterozygous carrier of the c.645del *TP53* variant. The proband's brother (III.1), who died at the age of 30 due to a pancreatic tumor, as well as the proband brother (III.4), deceased at the age of 70 due to thyroid carcinoma, did not receive any genetic testing. Two more proband's brothers (III.6) and (III.8) were referred to find health status and were found to have wild-type *TP53* alleles. The proband's brother (III.10), deceased at the age of 53 due to colorectal cancer, was himself a heterozygous carrier of the c.645del TP53 variant. Also, a 30-year-old deceased proband's sister (III.13) affected by both breast cancer and soft tissue sarcoma and his first female cousin (III.21) deceased at the age of 33 due to breast cancer were both heterozygous carriers of the c.645del *TP53* variant. Finally, the proband's niece (IV.19), who has a diagnosis of breast cancer at the age of 34, and the proband's nephew (IV.20), who was healthy, were found to have wild-type *TP53* alleles (Figure 2B).

Interestingly, other typical LFS tumors were described in the family pedigree. The proband's mother (II.2), who died at the age of 40, and his grandmother (I.2), who died at the age of 48, had breast and ovarian cancer, respectively. His half-sister (III.14), who had a 20-year-old deceased son due to osteosarcoma (IV.22) and an 18-year-old dead daughter due to spinocellular carcinoma (IV.21), was affected by a brain tumor and died at the age of 45. In addition, a brain tumor was found in the proband's nephew (IV.10), and the proband's first female cousin (III.22); they were deceased at the age of 42 and 45, respectively. All the proband's relatives affected by typical LFS cancers (I.2, II.2, III.14, III.22, IV.10, IV.21, IV.22, Figure 1) were deceased and did not receive any genetic testing.

## Discussion

Here, we describe for the first time a family with a novel and rare germline variant in the *TP53* gene responsible for LFS syndrome. Because the proband had a deceased affected son with osteosarcoma and a family having an aggregated history of typical LFS cancers, a mutational screening of the *TP53* gene was offered to him and his family members. Genetic testing revealed a heterozygous pathogenic germline *TP53* variant c.645del in the proband, his son, and three probands' relatives. At the protein level, the revealed c.645del variant causes a frameshift with the introduction of a premature stop codon within the p53 DNA binding domain (DBD) (p.Ser215Argfs^*^32). The DBD domain, located in the central part of the p53 protein, contains most of the LFS-associated missense variants (Bouaoun et al., 2016; AlHarbi et al., 2018). However, also frameshift pathogenic variants in the p53 DBD, such as c.685dup (p.Cys229Leufs^*^11), have been observed in a family with LFS (Ji et al., 2018).

The *TP53* frameshift variant identified here, not previously described in any database including HGMD, LOVD, and ClinVar, was classified as pathogenic based on the American College of Medical Genetics (ACMG) criteria (PVS1+PM2+PP1_Moderate) (Richards et al., 2015).

Notably, a duplication of two bases at the same region in the *TP53* gene (c.643_644dup), creating a frameshift starting at codon 215 (p.Ser215fs), was classified as pathogenic in the Clinvar database (accession ID: VCV000492747.1).

It is well-known that carriers of *TP53* pathogenic variants have an increased risk of early onset cancers, often recognized as LFS syndrome (Kharaziha et al., 2019; Yamamoto et al., 2020). Although LFS is a highly penetrant cancer syndrome, its penetrance in subjects with a *TP53* germline variant varies depending on the variant type (Varley et al., 2001; Bougeard et al., 2015). For example, the most severe variants of *TP53* associated with earlier tumor onset are the dominant-negative missense variants because of their ability to produce malfunctioning or non-functioning p53 tetramers (Frebourg et al., 2020). Instead, null variants, including frameshift variants, such as that described here, are mainly observed in families with typically adult cancers with a lower disease penetrance (Bougeard et al., 2015; Frebourg et al., 2020).

The novel inherited frameshift *TP53* variant described here results in a predicted truncated protein with a premature stop codon. There are two hypotheses explaining the consequence of this *TP53* frameshift variant on p53 protein function. One suggests that the modified mRNA could be unstable and, thus, degraded by non-sense-mediated mRNA decay. The other is that the modified mRNA could be transcribed into a truncated protein having a dominant-negative effect that disrupts the p53 tumor-suppressor function.

Here, we describe a family carrying a novel pathogenic germline variant of the *TP53* gene. This inherited frameshift variant is extremely rare because more than 70% of the pathogenic *TP53* variants in LFS families were observed to be missense variants (AlHarbi et al., 2018). This family case report highlights the importance of offering genetic counseling and genetic testing to all family members at high risk to be carriers of *TP53* inherited pathogenic variants.

## Data Availability Statement

The datasets presented in this article are not readily available due to ethical and privacy restrictions. Requests to access the datasets should be directed to the corresponding author.

## Ethics Statement

The patients/participants provided their written informed consent to participate in this study. Written informed consent was obtained from the individual(s) for the publication of any potentially identifiable images or data included in this article.

## Author Contributions

The study was conceived by RI, FP, and NP. VB, FF, FP, and EC performed the experiments. FP, EC, FF, FB, VB, and RI analyzed and interpreted the data. FP, RI, FT, FF, and NP wrote the manuscript. VB, NP, and FT supervised the project. All authors contributed to the article and approved the submitted version.

## Conflict of Interest

FP is employed by Tecnologica Research Institute and Marrelli Health. The remaining authors declare that the research was conducted in the absence of any commercial or financial relationships that could be construed as a potential conflict of interest.

## Publisher's Note

All claims expressed in this article are solely those of the authors and do not necessarily represent those of their affiliated organizations, or those of the publisher, the editors and the reviewers. Any product that may be evaluated in this article, or claim that may be made by its manufacturer, is not guaranteed or endorsed by the publisher.
