# Supplementary material for: Case Report: Identification of a Novel Pathogenic Germline TP53 Variant in a Family With Li–Fraumeni Syndrome
Source: Front Genet. 2021 Sep 1;12:734809. doi: 10.3389/fgene.2021.734809 (PMC8440986; doi:10.3389/fgene.2021.734809)
Supplement: Supplementary file 1 [file Data_Sheet_1.DOCX]

**Supplementary Methods**

*Next-generation sequencing (NGS)*

Genomic DNAs from probands and his relatives were extracted from blood samples after signing informed consent forms using the NLM DNA extraction kit (Nuclear Laser Medicine). We designed an Ion Ampliseq On-Demand panel to explore, using next-generation sequencing (NGS), the mutational status of the most frequently altered genes in cancers, including *TP53, BRCA2, BRCA1, ATM, CHEK2* and *PALB2* (Thermo Fisher Scientific). The designed panel covered the full coding exons plus padding regions of the above-described genes. The tumour panel consist of 296 amplicons with a range of 125-275 bp split into two primer pools (POOL1: 177 amplicons and POOL2: 179 amplicons). Libraries were constructed and purified on the Ion Chef Instrument according to the Ampliseq manufacturer’s instructions using 10.5 ng of template DNA for reaction. Template preparation was then automatically carried out on the Ion Chef Instrument. Subsequently, libraries with barcode were manually diluted to 30 pM and then re-loaded onto the Ion Chef for emulsion PCR, target enrichment, and loading onto the Ion S5 510 chip (Thermo Fisher Scientific). Sequencing data were generated by the Ion GeneStudio S5 System (Thermo Fisher Scientific). Germline variants were visually examined by IGV.

*Sanger sequencing*

Genomic DNAs were amplified by PCR using the forward and reverse primer binding to the exon6 of *TP53*. Standard PCR sequencing conditions were used (96°C for 10 s, 50°C for 5 s, 60°C for 2 min, 25 cycles). Amplicons were bidirectionally sequenced using Big Dye Terminator 1.1 on a SeqStudio Genetic Analyser (Thermo Fisher Scientific).
